# Vitamin A Status Modulates Epithelial Mesenchymal Transition in the Lung: The Role of Furin

**DOI:** 10.3390/nu16081177

**Published:** 2024-04-15

**Authors:** M. Teresa Cabezuelo, Luis Torres, Elena Ortiz-Zapater, Gerardo López-Rodas, M. Pilar Marín, Joaquín Timoneda, Juan R. Viña, Rosa Zaragozá, Teresa Barber

**Affiliations:** 1 Department of Physiology, University of Valencia, 46010 Valencia, Spain; tecabar@alumni.uv.es; 2 Centro Salud Safranar, Hospital Universitario Doctor Peset, 46017 Valencia, Spain; 3 Department of Biochemistry and Molecular Biology-IIS INCLIVA, University of Valencia, 46010 Valencia, Spain; luis.torres@uv.es (L.T.); elena.ortiz-zapater@uv.es (E.O.-Z.); gerardo.lopez@uv.es (G.L.-R.); joaquin.timoneda@uv.es (J.T.); juan.r.vina@uv.es (J.R.V.); teresa.barber@uv.es (T.B.); 4 Microscopy Unit IIS La Fe Valencia, 46009 Valencia, Spain; marin.marmue@gva.es; 5 Department of Human Anatomy and Embryology-IIS INCLIVA, University of Valencia, 46010 Valencia, Spain

**Keywords:** vitamin A deficiency, retinol, retinoic acid, lung, pulmonary disease, extracellular matrix, E-cadherin, N-cadherin, furin, epithelial–mesenchymal transition

## Abstract

Vitamin A deficiency (VAD) induced TGF-β hyperactivation and reduced expression of cell adhesion proteins in the lung, suggesting that the disruption of retinoic acid (RA) signaling leads to epithelial–mesenchymal transition (EMT). To elucidate the role of lung vitamin A status in EMT, several EMT markers and the expression of the proprotein convertase furin, which activates TGF-β, were analyzed in two experimental models. Our in vivo model included control rats, VAD rats, and both control rats and VAD rats, treated with RA. For the in vitro studies, human bronchoalveolar epithelial cells treated with RA were used. Our data show that EMT and furin are induced in VAD rats. Furthermore, furin expression continues to increase much more markedly after treatment of VAD rats with RA. In control rats and cell lines, an acute RA treatment induced a significant increase in furin expression, concomitant with changes in EMT markers. A ChIP assay demonstrated that RA directly regulates furin transcription. These results emphasize the importance of maintaining vitamin A levels within the physiological range since both levels below and above this range can cause adverse effects that, paradoxically, could be similar. The role of furin in EMT is discussed.

## 1. Introduction

Retinoids are a family of molecules that possess activity relative to vitamin A (all-*trans*-retinol) [1]. They exert major effects on numerous physiological processes, such as light transduction in vision, embryonic development, growth, immunity, cell differentiation and proliferation, tissue architecture, antioxidant function, redox signaling, and energy homeostasis [2,3,4,5,6,7,8]. Retinoids are also involved in several pathologies such as cardiovascular diseases, obesity, diabetes mellitus, respiratory diseases, osteoporosis, skin diseases, and cancer, among others [2,9,10,11].

The main biologically active retinoid metabolite of vitamin A is retinoic acid (RA), with all-*trans* RA being the predominant isomer in vivo. Multiple genes respond to RA signaling through both transcriptional and non-transcriptional mechanisms. Retinoid intracellular signaling occurs through two subfamilies of heterodimeric nuclear receptors, RA receptors (RARs), and retinoid X receptors (RXRs), each with three subtypes (α, β, and γ) and several isoforms. All-*trans* RA binds with high affinity to RAR but does not bind to RXR; on the contrary, the isomer 9-*cis* RA binds to both RARs and RXRs, although its effect in vivo remains elusive, given its low abundance in tissues [12]. To add complexity to retinoid signal transduction, RXRs form heterodimers with other members of the nuclear receptor family, including the peroxisome proliferator-activated receptor (PPAR), thyroid hormone, vitamin D, and orphan nuclear receptors [5,8,12,13].

Vitamin A is an essential fat-soluble micronutrient that must be provided in the diet, mainly in the form of retinyl esters but also all-*trans*-retinol, in products of animal origin, or as provitamin A (carotenoids) from fruits and vegetables. There are more than 50 carotenoids, with β-carotene being the most biologically significant and prevalent provitamin A. Since all these compounds are liposoluble they can be easily accumulated within the body. This storage allows for a pool in case of vitamin A deprivation, but it may also trigger vitamin A toxicity due to excessive accumulation [8,14,15,16]. Vitamin A is mainly stored in the liver but also in other organs, including the lung, where it plays an important role in ensuring retinoid signals for alveolus formation [8,17,18]. 

Vitamin A deficiency (VAD) is a significant health problem with important implications for global health policy. In fact, VAD causes impaired vision, reduced growth, and, even in its asymptomatic subclinical form, increases in the incidence of respiratory tract diseases and morbidity and mortality from various infections, especially in children. According to the World Health Organization, VAD constitutes, together with protein malnutrition, the most common nutritional disorder in the world [19]. 

Several studies have established that vitamin A is involved in the differentiation and maturation of the lung during intrauterine development, and it is also required to maintain alveolar architecture in adults [18,20,21,22]. Impaired retinoid signaling has been associated with histopathological changes in the parenchymal lung epithelium, which may predispose it to severe tissue dysfunction and respiratory disease. In this sense, VAD is associated with an increased risk of respiratory infections, emphysema, chronic obstructive pulmonary disease, pulmonary fibrosis, and lung cancer [8,21]. Some of the alterations found in lung tissue involve changes in the extracellular matrix (ECM) and modifications in proteins of the basement membrane (BM). It is known that RA regulates the expression of several components of the ECM such as collagens, laminins, fibronectin, elastin, or proteoglycans, and VAD may alter the content or distribution of the ECM [8,23]. 

Epithelial-to-mesenchymal transition (EMT) is a complex process in which polarized epithelial cells differentiate into contractile mesenchymal cells (more feasible for migration or invasion of other tissues) and involves modification of the ECM components. This EMT process takes place in many pulmonary diseases such as chronic obstructive pulmonary disease, fibrosis, and lung cancer [24,25]. Hallmarks of EMT include loss of E-cadherin and other cell adhesion proteins, together with an increase in mesenchymal markers, such as N-cadherin, vimentin, and α-smooth muscle actin (α-SMA), among others [26,27]. This differentiation switch can be promoted by several local stimuli, including the TGF-β signaling pathway, a powerful inducer of EMT. 

In a previous work, we suggested that VAD could trigger EMT in lung tissue. In fact, we have shown that in VAD rats, there are alterations in lung parenchymal architecture, associated with modifications in the ECM and the BM [23,28]. Collagens I and IV increased in the lungs of VAD rats, with an ectopic deposition of collagen I fibrils in the alveolar BM which doubles in thickness. In addition, chronic VAD activates TGF-β signaling, and there is oxidative stress and leucocyte infiltration in the lung. Furthermore, some cell adhesion proteins were reduced in VAD lungs [8]. All of these data suggest that disruption of endogenous RA signaling leads to the EMT process in the lung, and it could be an early stage common to several respiratory pathologies associated with this deficiency. 

To elucidate the role of vitamin A in the EMT process in the lung, we analyzed protein markers of EMT transition in vivo in control rats, vitamin A-deficient (VAD) rats, and in control and VAD rats treated with all-*trans*-RA (C + RA or VAD + RA groups). We have also analyzed the proprotein convertase furin, which regulates the levels of adhesion molecules and cleaves activating proproteins (i.e., TGF-β factor) and other cytokines, matrix metalloproteinases, and also glycoproteins on the cell surface of viral and bacterial pathogens that could increase their virulence (i.e., SARS-CoV-2) [29,30]. The effect of RA on EMT and furin expression was also evaluated in an in vitro model using cultured human lung cells supplemented with RA at different times.

Our data show that vitamin A status modulates EMT and furin expression in the lung and that this modulation can be, in part, produced by the direct control exerted by RA on furin expression. Our results emphasize the importance of tissue vitamin A levels, since both levels below and above this normal range can cause adverse effects, and these could, paradoxically, be similar. In the present study, we demonstrate that both vitamin A deficiency and RA treatment induce EMT and increase furin expression, which is associated with a variety of infectious and non-infectious diseases and has been proposed as a potential marker for various neoplasms, including lung cancer [29,30,31].

## 2. Materials and Methods

### 2.1. Antibodies

Primary antibodies against Furin (70393), Vimentin (5741), N-cadherin (14215), and β-Actin (4970) were purchased from Cell Signalling Technology (Danvers, MA, USA). Those against β-Catenin (ab32572), hsc70 (ab51052), and GAPDH (ab8245) were from Abcam (Cambridge, UK). Other primary antibodies used were: E-cadherin (80182) from BD Biosciences (Bedford, MA, USA) and α-SMA (14-9760-82) from Invitrogen™ (Waltham, MA, USA). All the HRP-conjugated antibodies were from DAKO (Nowy Sącz, Poland) and for immunofluorescence detection Alexa Fluor 488 anti-rabbit IgG (Invitrogen™) and Cy3 anti-mouse (Sigma-Aldrich, St. Louis, MO, USA) were used. For the ChIP assay, antibodies against RARα (sc-515796) and non-specific IgG (sc-2025) were used, both from Santa Cruz Biotechnology (Dallas, TX, USA).

### 2.2. Animals and Diets

All animal procedures were carried out in accordance with the NIH Guide for the Care and Use of Laboratory Animals. The Ethics Committee for Animal Research and Welfare at the University of Valencia and the GVA approved the protocol (2016/VSC/PEA/00130). Male-specific pathogen-free Wistar rats were made deficient in vitamin A by feeding a vitamin A-free diet. Briefly, pregnant rats (Charles River, Barcelona, Spain) were housed under standard conditions of light (12 h light cycle) and temperature (22 °C), and after delivery, dams with their litters were randomly divided into 2 groups. The control group (C group) received a complete purified diet (AIN-93G, ICN Biomedicals, Cleveland, OH, USA), which included vitamin A (all-*trans* retinyl palmitate 4000 IU/kg) [32]. The second group was fed the same diet from ICN Biomedicals but devoid of vitamin A (VAD group). 

After the lactation period, male pups were weaned to take their dam’s diet (control or VAD diet) until they were 60 days old (C group, VAD group). A third group of 60-day-old VAD rats were treated further with 10 daily intraperitoneal injections of 100 μg of all-*trans* RA (0.4 mg/Kg of all-*trans* RA, Sigma-Aldrich) in 100 μL of sunflower seed oil (which contains less vitamin traces than others), (VAD + RA group), or with an equal amount of vehicle. To summarize, our in vivo deficient model included:

- Offspring rats from control breeders were fed a control diet (control group, *n* = 6 males) until day 60 after delivery;
- Offspring rats from VAD diet-fed breeders were fed a VAD diet (VAD group, *n* = 18 males) until day 60 after delivery. At this point, two more groups were treated with RA or vehicle:
  ○ VAD rats were treated intraperitoneally with 100 μg of all-*trans* RA daily for 10 days (VAD + RA group, *n* = 6 males);
  ○ VAD rats were treated with 100 μL vehicle (sunflower oil) daily for 10 days (VAD + vehicle group, *n* = 6 males).

On the other hand, to study the effect of an acute RA overdose in control rats, a group of 60-day-old rats fed with a control diet were treated as follows:

- Ten daily intraperitoneal injections of 100 μg all-*trans* RA (0.4 mg/Kg of all-*trans* RA) in 100 μL of sunflower seed oil (C + RA group, *n* = 6 males).
- Ten daily intraperitoneal injections of 100 μL of sunflower seed oil (C + vehicle group, *n* = 6 males).

All the groups were pair-fed. Since the results obtained with the vehicle-treated groups did not differ from the corresponding VAD or C group, for simplicity, they are not shown. The sample sizes were determined using the G*Power 3.1 software. The calculation parameters were: (1) effect size = 0.8; (2) α = 0.05; (3) power = 0.85; (4) number of groups = 4; (5) numerator *df* = 3; and (6) denominator *df* = 20.

### 2.3. Sampling Procedure

Experiments were performed between 10:00 and 12:00 h. Rats were anesthetized with pentothal (50 mg/kg body weight, intraperitoneally), and blood samples and lung tissue were promptly harvested and stored at −80 °C until further use. 

### 2.4. Determination of Retinoids

Plasma retinol and lung retinoids were extracted as described [28], dissolved in methanol/ethanol (1:1, *v*/*v*), and measured by the isocratic HPLC method of Arnaud et al. [33]. A Novapak C-18 column (3.9 × 150 mm, Waters Technologies, Drinagh, Ireland) and a mixture of acetonitrile/dichloromethane/methanol (70:20:10), as eluent, were used.

### 2.5. Protein Extraction and Immunoblotting

Total protein was extracted in RIPA buffer (1.8 mM NaH_2_PO_4_, 8.4 mM Na_2_HPO_4_, 0.1% (*w*/*v*) SDS, 1.0% (*v*/*v*) TritonX 100, 0.1 M NaCl, 0.5% sodium deoxycholate, 1 mM PMSF) supplemented with protease and phosphatase inhibitors. Equal amounts of protein measured by a BCA protein assay kit were separated by SDS-PAGE gel electrophoresis and transferred onto nitrocellulose membranes (Amersham Protran^®^, Cytiva, Freiburg im Breisgau, Germany). Blots were developed by enhanced chemiluminescence reaction (ECL^TM^ Western blotting Detection reagents, Cytiva). Equal loading was confirmed by reprobing blots with β-actin, GAPDH, or hsc70 antibodies. To avoid membrane striping whenever possible, blots were cut and each strip was incubated with different antibodies to detect several proteins in the same membrane. Proteins from at least three different animals per experimental group were isolated and analyzed by Western blot. For protein quantification, Image J software 1.54 was used; each blot was normalized against β-actin, GAPDH, or hsc70, and plotted as fold change compared to controls.

### 2.6. RNA Isolation and Real-Time RT-qPCR Analysis

An RNAeasy Mini Kit (Qiagen, Hilden, Germany) was used to extract total RNA from lung tissue (3–5 rats per condition), followed by 10 min DNase I treatment (RNase-Free DNase set, Qiagen). At least 3–5 rats per condition were used. A bioanalyzer was used to assess RNA purity and integrity. RNA (385 ng) was reverse-transcribed to cDNA using the Transcriptor High Fidelity cDNA Synthesis kit (Roche, Basel, Switzerland) with a Random Hexamer Primer. cDNA products were amplified by qPCR using the LightCycler^®^ 480 SYBR Green I Master (Roche). All reactions were carried out in duplicate. Quantitative real-time PCR was run in the LightCycler^®^ 480 Instrument II (Roche). The genes that were analyzed were E-cadherin, N-cadherin, β-catenin, and furin, and β2-microglobulin (B2M) was the housekeeping gene selected for data normalization. The sequences for all primers used for cDNA amplifications are listed in Table 1.

To quantify gene expression, an analysis based on Ct values was carried out; for each sample and condition, the average of the duplicates was normalized with the average of the B2M housekeeping gene. Fold-change data were obtained using the delta–delta Ct method (2^−ΔΔCT^) where:

ΔCt = Ct (target) − Ct (housekeeping gene)

ΔΔCt = ΔCt (sample) − ΔCt (control)

### 2.7. Chromatin Immunoprecipitation Assay (ChIP Assay)

The ChIP procedure was performed as described previously [34]. In brief, lungs were surgically removed and fixed with 1% formaldehyde in PBS for 10 min. Samples were washed and homogenized in the presence of protease inhibitors (Sigma), filtered through a 500 mm pore nylon membrane, and centrifuged at 1500× *g* for 5 min. The pellet was resuspended cell lysis buffer (10 mM NaCl, 3 mM MgCl2, 30 mM sucrose, 10 mM EDTA, 0.5% Nonidet P-40, 10 mM Tris-HCl, pH 7), and incubated on ice for 15 min. Then, it was centrifuged at 3500× *g* for 5 min and the nuclear pellet was resuspended in nuclei lysis buffer (10 mM EDTA, 1% SDS, 50 mM Tris-HCl, pH 8.1) and stored at −80 °C.

Cross-linked chromatin was sonicated (3 cycles of 5 min sonication (30 s on, 30 s off) in a Bioruptor Plus instrument (Diagenode, Seraing, Belgium) and centrifuged at 14,000× *g* for 10 min. Supernatants were diluted 10-fold (165 mM NaCl, 0.01% SDS, 1.1% Triton X-100, 1.2 mM EDTA, 16.7 mM Tris–HCl, pH 8.0, supplemented with protease inhibitor cocktail (Sigma)). Aliquots from the diluted supernatants (equivalent to 50 μg DNA) were incubated under rotation for 2 h at 4 °C with Dynabeads Protein G (Invitrogen) and 2 μg of RARα or non-specific IgG antibodies. The chromatin fragment/antibody/protein G-Dynabead immune complexes were recovered and washed with low-salt buffer (140 mM NaCl, 1% Triton X-100, 0.1% sodium deoxycholate, 1 mM EDTA, 50 mM Tris-HCl, pH 8.0), high-salt buffer (500 mM NaCl, 1% Triton X-100, 0.1% sodium deoxycholate, 1 mM EDTA, Tris-HCl 50 mM, pH 8.0), LiCl buffer (250 mM LiCl, 0.5% NP-40, 0.5% sodium deoxycholate, 1 mM EDTA, Tris–HCl 10 mM, pH 8.0), and finally with TE buffer. The immunoselected chromatin was eluted by adding 50 μL of elution buffer (EDTA 10 mM, SDS 1%, 50 mM Tris–HCl), vortexing, and incubating for 10 min at 65 °C.

The resulting 100 μL fraction (IP fraction) was incubated at 65 °C overnight to reverse formaldehyde cross-links in the presence of RNAase 40 μg/mL, and then proteins were digested by proteinase K (0.4 mg/mL). An aliquot of the cross-linked chromatin was treated as above, but in the absence of antibody (NA fraction), and the first supernatant was saved as the Input fraction. The DNA (from IP, NA, and Input samples) was purified with a PCR purification kit (Qiagen) and used for qPCR analysis to determine the binding of RARα at the promoter region of the furin gene using primers, as shown in Table 2. The MMP-9 gene was used as a positive control since we had previously described that RARα binds to the MMP-9 promoter upon RA treatment [35]. The entire experiment was performed with lung samples from three different rats per condition.

### 2.8. Cell Culture and Treatments

Two human cell lines were used in this study. BEAs non-tumoral bronchoepithelial cells were a generous gift from Dr. Julio Cortijo’s lab and were cultured at 37 °C in a CO_2_ incubator in RPMI Medium (Gibco, Thermo Fisher, Waltham, MA, USA) supplemented with 10% FBS (Biowest, Nuaillé, France), 1% penicillin/streptomycin (Gibco), and L-glutamine (G7513, Sigma-Aldrich) under standard conditions. On the other hand, A549 cells were purchased from American Type Culture Collection (ATCC) including certificate of analysis and mutation sequencing data. These are malignant cells from basal alveolar epithelia and are cultured under the same conditions but with DMEM (Gibco) as the culture media. 

For experiments, cells were plated at a density of 8 × 10^5^ cells/well and maintained in asynchronous culture under standard conditions. When cells reached confluency, RA (Sigma-Aldrich, R2625) was added at a final concentration of 5 μM for 24 h or 48 h. Since bovine serum contains retinoids, cells were starved in a serum-free medium prior to the experiments. Only low-passage cells (8–12 passages) were used for the studies and after RA treatment cell viability was assessed prior to collecting the samples. All experiments shown were performed in triplicate. 

### 2.9. Immunofluorescence Analysis

Cells were cultured onto 13 mm Ø borosilicate Cover Glass (631-0149, VWR, Radnor, PA, USA) fixed in PFA 4% for 15 min, permeabilized with Triton TX-100 0.1% in TBS, blocked in 2% BSA, and incubated with the indicated primary antibodies overnight at 4 °C. The proper secondary antibody was used for detection. Nuclei were counterstained with DAPI (Invitrogen^TM^). Pictures were acquired on a Leica TCS-SP 2 confocal microscope.

### 2.10. Statistical Analysis

The results are represented in the figures and tables as the mean ± SD of at least three independent experiments. Statistical analyses were performed using the GraphPad Prism software (v. 10.2.2). For each experiment, an outlier test was performed, and statistical outliers were removed from the raw data. Finally, differences between groups were determined by a student’s *t*-test followed by a post hoc Bonferroni test. Significant differences are indicated on each figure legend, being * *p* ≤ 0.05; ** *p* ≤ 0.01 *** *p* ≤ 0.001, or **** *p* ≤ 0.0001.

## 3. Results

### 3.1. Retinoid Levels in Plasma and Lung Tissue

Prior to investigating the effects of vitamin A deprivation in lung tissue, retinoid levels were measured in the plasma and lung of control rats (Control), VAD rats (VAD), and deficient rats treated with an acute dose of RA (VAD + RA) (Table 3). As already reported, those rats fed a vitamin A-free diet from birth (through the dam’s milk) (VAD group) had plasma retinol concentrations that were less than 5% of the concentration found in 60-day-old controls (C) and this value did not increase with the all-*trans* RA treatment (VAD + RA). All-*trans* retinyl esters were not detectable in the plasma in any group [28].

In parallel, retinoid levels were also measured in the lung tissue; the amount of retinol dropped more than 95% in both VAD and VAD + RA lungs compared to control tissues. Furthermore, the levels of retinyl esters, the main retinoids found in the lung, were also significantly lower in VAD and VAD + RA animals than those in the control group. The results obtained in the VAD group treated with vehicle instead of RA did not differ from the VAD group. Our model of chronic vitamin A deficiency completely depleted retinol in the plasma and induced long-time vitamin A reduction in lung tissue.

### 3.2. Vitamin A Status and EMT Markers

**Figure 1:**
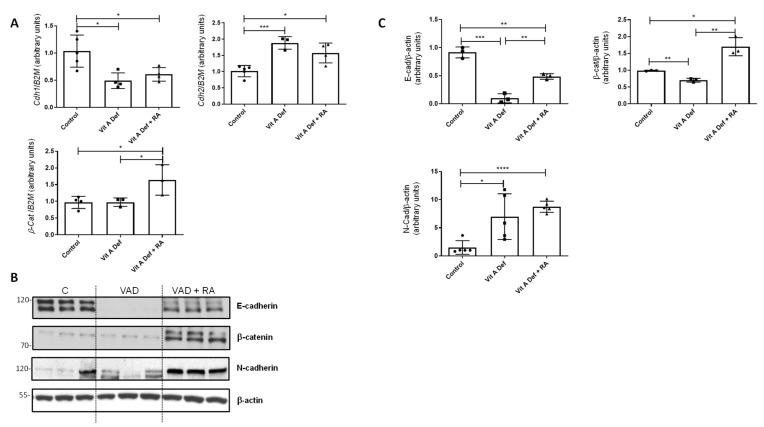
Vitamin A status and EMT markers in the lung. (**A**) RT-qPCR was performed to analyze the mRNA expression of EMT markers Cdh1, Cdh2, and β-catenin in control rats, vitamin A-deficient rats (VAD), and vitamin A-deficient rats treated with retinoic acid (RA). The data used in the statistical analyses and histograms are the mean ± SD (n = 4–5 for each group). (**B**) Protein levels of different EMT markers (E-Cadherin, N-Cadherin, and β-catenin) were studied by Western blot in lungs from control (C), vitamin A-deficient (VAD), and vitamin A-deficient rats treated with RA (VAD + RA). (**C**) Graphs showing the Western blot quantification. Data (n ≥ 3) were quantified, normalized with β-actin, and plotted as mean fold ± SD vs. control. (**A**,**C**) significant results are shown * *p* ≤ 0.05; ** *p* ≤ 0.01 *** *p* ≤ 0.001 or **** *p* ≤ 0.0001.

EMT is characterized by the loss of epithelial markers (E-cadherin and β-catenin) concomitant with an induction of mesenchymal markers (N-cadherin and vimentin). In our vitamin A-deficient model, there was a significant change in the expression of *cdh1* (E-cadherin) and *cdh2* (N-cadherin), both EMT markers (Figure 1A). Indeed, there was an EMT switch: *Cdh1* decreased in the lungs after vitamin A deficiency (VAD) whilst *Cdh2* levels increased statistically. On the other hand, *β-catenin* mRNA levels did not change in VAD lungs, but its expression significantly increased after RA treatment (VAD + RA) (Figure 1A). β-catenin is a transcriptional coactivator known to be upregulated in TGF-β-induced EMT [36].

At the protein levels, we found similar results (Figure 1B); the expression of E-cadherin decreased in VAD lungs compared to controls, whereas the expression of N-cadherin increased in VAD (Figure 1B,C for quantification). Treatment of deficient rats with RA (VAD + RA) increased E-cadherin and β-catenin protein levels compared to the VAD group. 

### 3.3. Furin Expression in Lungs

**Figure 2:**
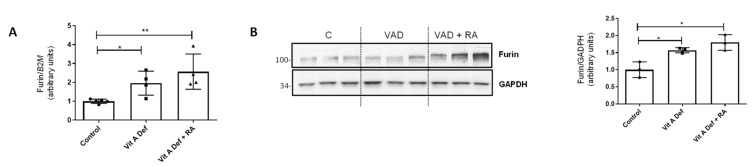
Vitamin A status and furin expression in the lung. (**A**) RT-qPCR showing mRNA levels of proconvertase furin in control rats, vitamin A-deficient rats, or vitamin A-deficient rats treated with retinoic acid (RA) (n = 4–5 per group). (**B**) Western blot analysis of furin in lungs from control (C), vitamin A-deficient (VAD), and vitamin A-deficient rats treated with RA (VAD + RA) (n = 3). GAPDH was used for normalization and the graph on the right represents quantification of the blots of three independent cultures. For both panels, histograms represent the mean ± SD, and results that were significantly different were * *p* ≤ 0.05; ** *p* ≤ 0.01.

Furin is a proprotein convertase that acts through limited proteolysis and converts target proproteins into bioactive proteins and peptides. Furin has been involved in the activation of molecules that promote cell proliferation, vascularization, or tissue migration and invasion [29,31]. In the EMT context, it has been described that furin can activate TGF-β acting as an EMT inducer [37]. Based on previous, unpublished results, we wanted to explore the relationship between EMT produced by vitamin A deficiency and furin. First of all, furin mRNA levels were analyzed in our experimental groups (Control, VAD, and VAD + RA). As shown in Figure 2A, the expression of furin increased in VAD lungs, but unexpectedly, RA treatment did not restore furin expression to the control values, as it continued to increase in VAD rats treated with RA. Western blot analysis (Figure 2B) showed that furin protein levels have a similar pattern to that found in the qPCR analysis.

### 3.4. Retinoic Acid Addition In Vitro in Human Lung Cells

**Figure 3:**
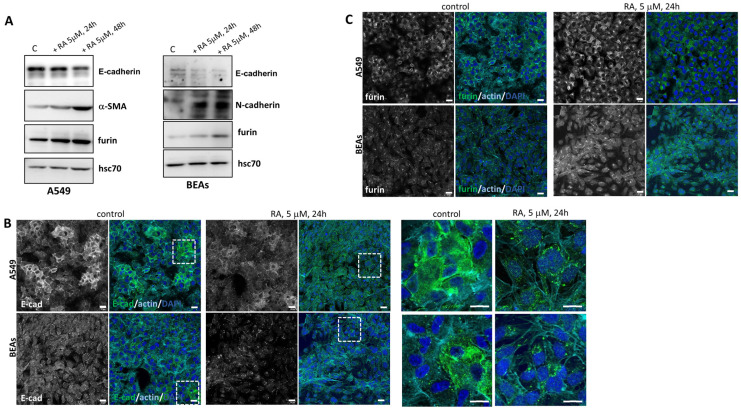
Effect of RA addition in vitro in human lung cell lines. (**A**) Western blot analysis showed the expression of different EMT markers and furin in two cell lines (A549 and BEAs) after incubation with 5 μM RA at 24 h and 48 h. Hsc70 was used as a loading control. A representative image is shown (n = 3 independent cultures). (**B**) Localization of E-cadherin (green), detected by immunofluorescence staining in A549 and BEA cells after 5 μM RA treatment for 24 h. Zoom has been made around the dotted lines and magnified images are shown on the right side. (**C**) Representative IF analysis of furin (green) in lung cell lines (A549 and BEAs) after 24 h of RA incubation (5 μM). In both (**B**,**C**), nuclei were stained with DAPI and F-actin with phalloidin (cyan blue). Scale bars 20 μM and 60 μM for zoom images in (**B**).

To further analyze these previous results, we moved to an in vitro model and treated two human cell lines with RA: the A549 lung adenocarcinoma cells and BEAs, which are bronchial epithelial immortalized cells. As observed in Figure 3A, there was a decrease in the epithelial marker E-cadherin in both cell lines after the addition of 5 μM RA. Moreover, there was also an increase in mesenchymal markers such as α-SMA and N-cadherin in A549 and BEAs cells, respectively. These results showed once more that vitamin A levels have a profound effect on EMT markers. The changes in protein levels were time-dependent in both cell lines, as RA incubation for 48 h showed changes that were more evident than those observed at 24 h. The effect of RA addition was also studied using immunofluorescence. Consistent with an EMT effect, E-cadherin was internalized after RA addition, both in A549 and BEAS cell lines (Figure 3B). 

These changes observed in the protein levels of different EMT markers after RA treatment were also dose-dependent. Indeed, A549 cells were treated with increasing concentrations of RA (0.1–10 μM) for 48 h, and changes in α-SMA were observed with 5 μM RA, whilst N-cadherin protein levels increased with 2.5 μM RA (Supplementary Figure S1).

We next studied changes in furin levels after RA addition. As shown by Western blot (Figure 3A) and by confocal microscopy (Figure 3C), 5 μM RA treatment increased levels of furin in a time-dependent manner. It can be concluded that the in vitro addition of RA to lung cell lines produces changes in EMT markers accompanied by a clear increase in furin protein levels. In agreement with our in vivo results, a relationship between EMT, vitamin A, and furin levels has also been observed in vitro.

### 3.5. Retinoic Acid Addition In Vivo to Control Rats

**Figure 4:**
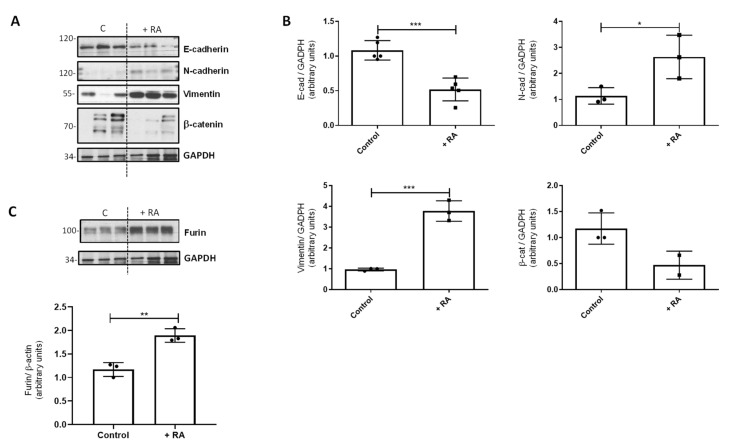
Effect of RA addition in vivo on EMT markers. (**A**) Western blot analysis to study the levels of different EMT markers in vivo in control rats after RA treatment (+RA group). GAPDH was used as a loading control. (**B**) Quantification of the Western blots is shown in panel (**A**). Data (n ≥ 3 different animals) were quantified, normalized with GAPDH, and plotted as mean fold ± SD vs. control. (**C**) Western blot analysis of furin in control rats after RA treatment (+RA group) (n = 3); GAPDH was used for normalization and quantification (mean fold ± S.D. vs. control). For all quantifications, asterisks indicate significant differences: * *p* ≤ 0.05; ** *p* ≤ 0.01, or *** *p* ≤ 0.001.

We also analyzed the effect of RA addition in vivo in control rats. As happened with lung cell lines, the addition of RA to control rats also produced changes in EMT markers in lung tissue, decreasing the protein levels of E-cadherin and β-catenin and increasing the levels of N-cadherin and vimentin (Figure 4A,B). As expected, furin followed the same pattern as EMT markers, as RA addition also increased the levels of this proconvertase (Figure 4C). 

### 3.6. Chromatin Immunoprecipitation Assay

**Figure 5:**
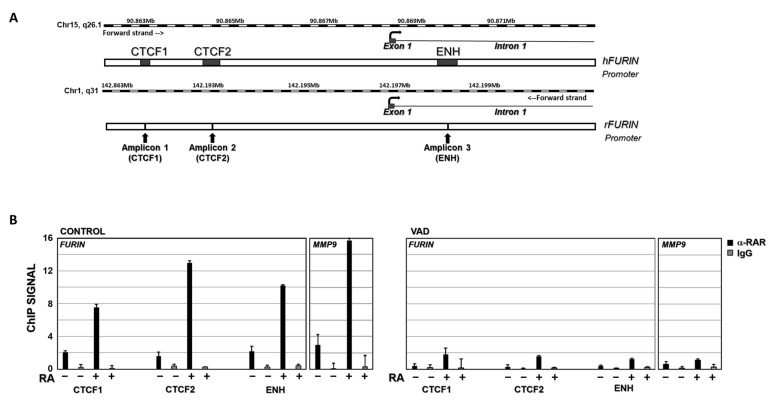
RARα ChIP assay on furin promoter. (**A**) Potential binding sites for RAR and/or RXR nuclear receptors in the furine promoter region. CTF1, CTF2, and Enhancer (ENH) indicate 3 regions where most transcriptional factor binding sites that modulate gene transcription are concentrated. (**B**) ChIP data analysis of RARα binding to furin and MMP-9 genes in control rats (RA−) and control rats injected intraperitoneally with retinoic acid (RA+) (left panel, CONTROL group). The same analysis was carried out in vitamin A-deficient rats (RA−) and vitamin A-deficient rats injected intraperitoneally with retinoic acid (RA+) (Right panel, VAD). Chromatin was immunoprecipitated with an anti-RARα antibody (black bars) or a nonrelated antibody anti-IgG as a negative control (grey bars). Data are the result of three independent experiments and bars represent mean ± SD.

To elucidate the relationship between vitamin A status and furin, we analyzed whether retinoic acid could directly regulate furin transcription by binding to the corresponding gene promoter. To confirm this hypothesis, a ChIP assay was performed. The ENSEMBLE database (https://www.ensembl.org, accessed on 6 July 2022) was used to search for regulatory regions in the furin gene promoter. In the available human data, up to three regions, named CTCF1, CTCF2, and Enhancer (ENH), are potential regulatory regions, where transcription factors would bind to modulate gene transcription (Figure 5A). Furthermore, analysis of the human and rat furin promoters in the EPD Eukaryotic Promoter Database (https://epd.epfl.ch/, accessed on 6 July 2022) indicates the presence of potential binding sites for two nuclear receptors, retinoid X receptor (RXR) and retinoid acid receptor (RAR), in these regions of the gene.

The results of ChIP assays showed that the nuclear factor RARα can bind to all three regions in the furin gene promoter, where potential RAR/RXR binding sites are located (https://epd.expasy.org/epd/, accessed on 6 July 2022). In control rats, the ChIP signal was 2.0, 1.6, and 2.2 for the CTCF1, CTCF2, and ENH regions, respectively (Figure 5B, left panel, CONTROL RA−). When this group was injected intraperitoneally with RA (RA+), the ChIP signal was significantly increased compared with untreated controls (RA−). Indeed, these increases were 3.7-fold for CTCF1, 8.3-fold for CTCF2, and 4.7-fold for ENH binding sites compared to the untreated control group (Figure 5B, left panel, CONTROL RA+). In ChIP assays using a non-specific IgG as a negative control, the corresponding signal was virtually undetectable. In contrast, in the ChIP assay of the MMP-9 gene, a positive control in which we had previously analyzed the binding of RARα to its promoter [35], the ChIP signal increased from 2.9 to 15.7 in RA-treated rats versus untreated controls (Figure 5B, left panel, MMP-9 gene). This result demonstrates that RA can modulate furin expression by binding to its gene promoter. 

Considering that vitamin deficiency may therefore modulate furin expression (Figure 2), we sought to elucidate the interaction of RARα with the furin promoter in our chronic vitamin A deprivation (VAD) model. As shown in Figure 5B, in ChIP assays with the VAD group (right panel, RA−), the ChIP signal was approximately 5-fold lower for the CTCF1, CTCF2, and ENH binding sites (VAD, RA−) compared to control rats fed a complete diet (CONTROL, RA−). On the other hand, when VAD rats were injected intraperitoneally with RA, the ChIP signal increased 4.1-fold, 4.8-fold, and 2.9-fold, for the CTCF1, CTCF2, and ENH binding sites, respectively (VAD, RA+), relative to untreated deficient rats (VAD, RA−) (Figure 5B, right panel); however, the ChIP signal remained significantly lower in the VAD group compared to control rats. Finally, the negative control with non-specific IgG gives undetectable signals. In contrast, the positive control used, the MMP-9 gene, gives a significant increase in VAD rats when treated with RA, although this is much more limited than in control rats.

Taken together, these data indicate that the furin gene is regulated by the binding of the retinoic acid receptor to its promoter and that its activation is dependent on the relative concentration of RA in the rats.

## 4. Discussion

Several studies have shown the important role played by retinoids in pulmonary physiology, function, and pathophysiology [8,17,18,19,20,21,22]. Previously, we had already found that VAD in vivo is associated with reduced protein levels of E-cadherin and β-catenin and increased levels of N-cadherin, together with increased expression of collagens I and IV and altered activities of matrix metalloproteinases [8,23,28,38]. All of these are changes in key markers of EMT. This process, in which differentiated epithelial cells acquire mesenchymal capabilities, is accompanied by ECM changes, rendering a migratory and invasive phenotype. In fact, EMT is an early-stage common factor in several respiratory pathologies associated with pulmonary fibrosis development and lung cancer [24,26,36,39,40,41]. In the present work, we have evaluated, in vivo and in vitro, the role of pulmonary vitamin A status on EMT markers and on furin expression, a protein proconvertase that regulates the levels of adhesion molecules and activates proteolytically matrix metalloproteinases, TGF-β factor, and other cytokines and receptors [29,31]. In agreement with all these functions, several studies suggest that furin promotes EMT in different cell types [42,43]. 

Data from Western blotting and qPCR confirmed that in our VAD model, there was a switch from E-cadherin to N-cadherin, compatible with an EMT in lung tissue from deficient rats. In other tissues it has been demonstrated that vitamin A plays a role in the inhibition of EMT [44]; surprisingly, in our model, treatment with all-*trans*-RA to these deficient animals did not completely restore gene expression levels to control values. These results are in agreement with previously published data in which components from the basement membrane or extracellular matrix were affected by vitamin A deficiency and were not completely recuperated after RA treatment [23,28]. In this sense, it is important to highlight that VAD rats are under oxidative stress, and in VAD + RA-treated rats, oxidative stress remains elevated [28], which could explain the results observed in our model. On the other hand, although the information provided in the literature on the effect of RA on TGF-β1 is controversial, in previous studies we have shown that VAD lungs showed increased active TGF-β1 [28], which is an EMT-inducer [26,39,40,45].

To further unveil the molecular mechanisms through which RA exerts its effects on lung tissue, we also analyzed the proconvertase furin in our VAD model. Furin is a ubiquitously expressed calcium-dependent endoprotease, which cleaves activating proproteins at their paired basic residues. Its mammalian substrates are a broad variety of precursor proteins including TGF-β, receptors, adhesion molecules, matrix metalloproteinases which could alter ECM, and cell surface glycoproteins of numerous viral and bacterial pathogens. A growing amount of evidence has suggested that altered furin expression and abnormal cleavage of its substrates may have a crucial role in several pathophysiological processes such as inflammation, neurodegeneration, cancer, or even viral infections [29,30,31]. Moreover, it has been recently published that furin could be modulated by the profibrotic cytokine TGF-β in differentiated human bronchial epithelial cells [46]. Other reports suggest that there is a positive feedback loop between furin and TGF-β1, resulting in an amplified TGF-β response [29,47,48]. In our model, the expression of the proconvertase furin increased in VAD lungs, and RA treatment did not restore furin expression to control values, as it was more markedly increased in VAD rats treated with RA (Figure 2). All together, these results pinpoint that RA treatment was not enough to avoid EMT or even to completely rescue those changes observed within the ECM in deficient lungs [23]. It seems that several factors still induced in VAD + RA rats, such as the remaining TGF-β signaling, increased furin levels, or oxidative stress, could be responsible for not having a complete recovery under RA treatment. 

In order to point out the effect of RA in EMT and furin expression, we used two different models in which RA was added in vivo and in vitro. On the one hand, control rats in vivo were injected with an acute overdose of RA, while the in vitro model consisted of the addition of RA to human cell lines for 24–48 h. Both approaches showed that the excess of RA also induces EMT markers (Figure 3 and Figure 4). In this sense, in vivo treatment of control rats with RA significantly decreased E-cadherin and β-catenin, whereas N-cadherin and vimentin protein levels were clearly increased. In vitro, incubation with 5 μM of RA decreased the epithelial marker E-cadherin and increased the levels of α-SMA and N-cadherin, consistent with the EMT process, and these changes were time- and dose-dependent. Regarding furin, the protein levels were increased in both experimental models, accompanying the EMT processes observed when an excess of RA was used. 

All in all, the results presented herein have shown that furin levels increase in a model of chronic VAD, but also under RA treatment. So, we wondered whether RA could somehow be modulating furin expression. To elucidate this possibility, a ChIP assay was performed. Our results clearly showed that in vivo injection of RA to control or deficient rats induced a significant increase in the binding of RARα to the furin promoter (Figure 5). Accordingly, furin expression and protein levels increased in both control and VAD rats treated with RA (Figure 2 and Figure 4C). The performed ChIP assay suggests that this modulation can be produced by the direct control exerted by RA on furin expression. However, the increased expression of furin in deficient rats (VAD) with altered RA signaling cannot be explained by the binding of RARα to the promoter. One could hypothesize that furin levels could be modulated by other signaling factors or other molecules different to RA. Interestingly, TGFβ, which is elevated in VAD rats [28], increases the expression and activity of furin, thus establishing a feedback loop that could justify the increase of this protein in retinoid deficiency [29,46,47,48].

**Figure 6:**
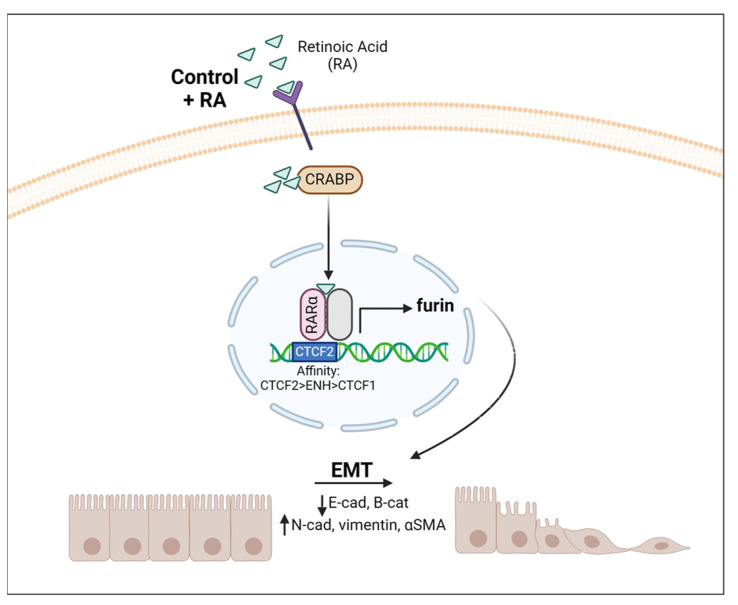
Vitamin A modulates Furin expression and EMT in the lung. Schematic representation of RA signaling via the RAR–RXR pathway when RA is added. This induces a transcriptional change within the nucleus, increasing furin expression that proteolyzes target proteins, triggering EMT. CRAPB: cellular retinoic acid binding protein.

In conclusion, our study shows that vitamin A status modulates EMT as well as furin expression in the lung. Furin levels seem to follow the same pattern as the EMT markers analyzed in this article, i.e., furin levels increase with vitamin A deficiency in vivo, but also with RA addition in vivo and in vitro. This is in accordance with the role of furin in proteolytic activation of TFG-β, a key regulator of EMT [39,40,42,45]. The performed ChIP assay suggests that this modulation can be, in part, produced by the direct control exerted by RA on furin expression (Figure 6).

As previously mentioned, vitamin A is involved in the proliferation and maintenance of epithelial cells and it is well known that the lack of vitamin A has serious consequences in lungs [8,17,18,20,21,22]. Indeed, several findings have suggested that higher intakes of vitamin A may beneficially influence lung growth and hence optimal lung function attainment [49]. Nevertheless, this relationship between dietary vitamin A and respiratory outcomes is not clear, with some trials showing the benefits of supplementation while others point to the toxicity of this molecule. Our results are another clear and relevant example that may demonstrate, at least in part, the adverse effects and conflicting results in lung studies in patients with vitamin A supplementation. In this context, several references have shown that vitamin A intake enhances TNF-α and is also associated with oxidative stress and increased proinflammatory cytokines in lung tissue; thus, a retinoid overdose may have a negative impact on maintaining tissue integrity [50,51,52,53,54]. In addition, there are conflicting results that correlate vitamin A with increased carcinogenic risk [55,56,57].

## 5. Conclusions

Our results emphasize the importance of maintaining vitamin A tissue levels within a normal range since both levels below and above this range can cause adverse effects, and these could paradoxically be similar [50,58]. In the present study, we demonstrate that both vitamin A deficiency and RA treatment induce EMT and increase expression of furin proconvertase, which is associated with a variety of infectious and non-infectious diseases and has been proposed as a potential marker for various neoplasms, including lung cancer [31]. Therefore, the preventive and therapeutic values of vitamin A and its derivatives in different processes, especially in certain types of cancers, should be carefully reviewed.

## Figures and Tables

**Table 1:** Primers Sequences.

Sequence Name	Sequence Forward	Sequence Reverse
*Furin*	ACTGCCCACCCTATCAAATC	CCAAACCCAGTCCCAAGATAA
*Cdh1* (E-cadherin)	GGGTTGTCTCAGCCAATGTT	CACCAACACACCCAGCATAG
*Cdh2* (N-cadherin)	GAGAGGAAGACCAGGACTATGA	TCTCGTCTAGCCGTCTGATT
*β-catenin*	CATATGCGGCTGCTGTTCTA	CCGAAAGCCGTTTCTTGTAG
*B2M*	CGAGACGATGTATATGCTTGC	GTCCAGATGATCAGAGCTCCA

**Table 2:** Primers used for ChIP analysis by qPCR.

Target Region	Primers	
	Forward	Reverse
*Furin* promoter		
CTCF1	TGTCCATCATCACCAGAGCT	CCCTCTTCTGGTGTGTCTGT
CTCF2	ACTGGAAAGTTACCGCCTGA	ACGTCACCATCTAGCTCCAG
ENHANCER	GCTTGGCTTGTGACTAGTCG	ACCAAGGTGAGGCTGAATCA
*MMP-9* promoter	GTGAACACGGTGGCTGAAA	CAGGCTCTTTGAAGCAGGATT

**Table 3:** Concentrations of retinol and retinyl esters in plasma and lungs of control, VAD, and VAD + RA rats.

	Control	VAD	VAD + RA
Plasma (μM)			
All-*trans* retinol	1.46 ± 0.26	0.06 ± 0.01 ****	0.05 ± 0.01 ****
All-*trans* retinyl esters	ND	ND	ND
Lung (nmol/g of tissue)			
All-*trans* retinol	1.40 ± 0.25	0.04 ± 0.01 ****	0.05 ± 0.01 ****
All-*trans* retinyl esters	2.66 ± 0.35	0.007 ± 0.02 ****	0.05 ± 0.03 ****

Values are expressed as mean ± S.D (n = 6); **** *p* < 0.0001 vs. control group.

## Data Availability

Data are contained within the article and Supplementary Materials.

## Supplementary Materials

The following supporting information can be downloaded at: https://www.mdpi.com/article/10.3390/nu16081177/s1, Figure S1: Effect of RA addition in vitro in A549 human lung cell lines.
