# Supplementary material for: Vitamin A Status Modulates Epithelial Mesenchymal Transition in the Lung: The Role of Furin
Source: Nutrients. 2024 Apr 15;16(8):1177. doi: 10.3390/nu16081177 (PMC11053499; doi:10.3390/nu16081177)
Supplement: Supplementary file 1 [file nutrients-16-01177-s001.zip › nutrients-2949843-supplementary.pdf]

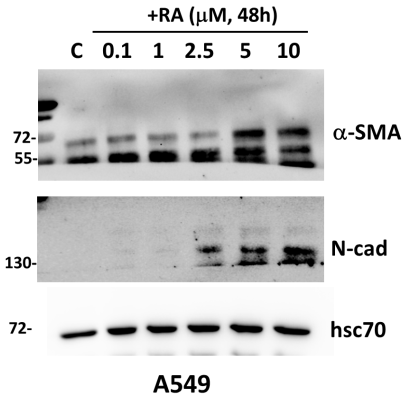

Supplementary Figure S1. Effect of RA addition in vitro in A549 human lung cell lines. Western blot analysis showed the expression of the EMT markers α-SMA and N-cadherin after incubation with increasing concentrations of all-trans RA for 48 h. hcs70 was used as loading control.
